# Reassessing Google Flu Trends Data for Detection of Seasonal and Pandemic Influenza: A Comparative Epidemiological Study at Three Geographic Scales

**DOI:** 10.1371/journal.pcbi.1003256

**Published:** 2013-10-17

**Authors:** Donald R. Olson, Kevin J. Konty, Marc Paladini, Cecile Viboud, Lone Simonsen

**Affiliations:** 1 New York City Department of Health and Mental Hygiene, New York, New York, United States of America; 2 Fogarty International Center, National Institutes of Health, Bethesda, Maryland, United States of America; 3 Department of Global Health, George Washington University, Washington, D.C., United States of America; Imperial College London, United Kingdom

## Abstract

The goal of influenza-like illness (ILI) surveillance is to determine the timing, location and magnitude of outbreaks by monitoring the frequency and progression of clinical case incidence. Advances in computational and information technology have allowed for automated collection of higher volumes of electronic data and more timely analyses than previously possible. Novel surveillance systems, including those based on internet search query data like Google Flu Trends (GFT), are being used as surrogates for clinically-based reporting of influenza-like-illness (ILI). We investigated the reliability of GFT during the last decade (2003 to 2013), and compared weekly public health surveillance with search query data to characterize the timing and intensity of seasonal and pandemic influenza at the national (United States), regional (Mid-Atlantic) and local (New York City) levels. We identified substantial flaws in the original and updated GFT models at all three geographic scales, including completely missing the first wave of the 2009 influenza A/H1N1 pandemic, and greatly overestimating the intensity of the A/H3N2 epidemic during the 2012/2013 season. These results were obtained for both the original (2008) and the updated (2009) GFT algorithms. The performance of both models was problematic, perhaps because of changes in internet search behavior and differences in the seasonality, geographical heterogeneity and age-distribution of the epidemics between the periods of GFT model-fitting and prospective use. We conclude that GFT data may not provide reliable surveillance for seasonal or pandemic influenza and should be interpreted with caution until the algorithm can be improved and evaluated. Current internet search query data are no substitute for timely local clinical and laboratory surveillance, or national surveillance based on local data collection. New generation surveillance systems such as GFT should incorporate the use of near-real time electronic health data and computational methods for continued model-fitting and ongoing evaluation and improvement.

## Author Summary

In November 2008, Google Flu Trends was launched as an open tool for influenza surveillance in the United States. Engineered as a system for early detection and daily monitoring of the intensity of seasonal influenza epidemics, Google Flu Trends uses internet search data and a proprietary algorithm to provide a surrogate measure of influenza-like illness in the population. During its first season of operation, the novel A/H1N1-pdm influenza virus emerged, heterogeneously causing sporadic outbreaks in the spring and summer of 2009 across many parts of the United States. During the autumn 2009 pandemic wave, Google updated their model with a new algorithm and case definition; the updated model has run prospectively since. Our study asks whether Google Flu Trends provides accurate detection and monitoring of influenza at the national, regional and local geographic scales. Reliable local surveillance is important to reduce uncertainty and improve situational awareness during seasonal epidemics and pandemics. We found substantial flaws with the original and updated Google Flu Trends models, including missing the emergence of the 2009 pandemic and overestimating the 2012/2013 influenza season epidemic. Our work supports the development of local near-real time computerized syndromic surveillance systems, and collaborative regional, national and international networks.

## Introduction

Influenza remains a paradox for public health: While influenza epidemics are expected seasonally in temperate climates, their exact timing and severity remain largely unpredictable, making them a challenge to ongoing preparedness, surveillance and response efforts [1]. Surveillance efforts for influenza seek to determine the timing and impact of disease through characterizing information on reported illnesses, hospitalizations, deaths, and circulating influenza viruses [2]. Since establishment of the first computerized disease surveillance network nearly three decades ago [3]–[5], the use of information and communications technology for public health disease monitoring has progressed and expanded. During the last decade, the use of electronic syndromic surveillance systems have allowed for automated, detailed, high volume data collection and analysis in near-real time [6]–[9]. In parallel, novel approaches based on influenza-related internet search queries have been reported to yield faster detection and estimation of the intensity of influenza epidemics [10]–[16]. The public health utility of such systems for prospective monitoring and forecasting of influenza activity, however, remains unclear [17]–[21], particularly as occurred during the 2009 pandemic and the 2012/2013 epidemic season [22]–[24].

In November 2008, Google began prospectively monitoring search engine records using a proprietary computational search term query model called Google Flu Trends (GFT) to estimate national, regional and state level ILI activity in the United States (US) [12]. The goal of GFT was to achieve early detection and accurate estimation of epidemic influenza intensity [13]. The original GFT model was built by fitting linear regression models to weekly counts for each of the 50 million most common search queries, from the billions of individual searches submitted in the US between 2003 and 2007 [13]. An automated query selection process identified the exact text searches that yielded the highest correlations with national and regional influenza-like-illnesses (ILI) surveillance in the US during the period of model fitting; the top scoring 45 search terms constituted the original GFT ILI search definition.

The GFT search algorithm was revised in the autumn of 2009, following the emergence and rapid spread of the pandemic A/H1N1pdm09 influenza virus in the US, which had gone wholly undetected by the GFT system. The updated GFT model used surveillance data from the first 20 weeks of the pandemic and a qualitative decision process with less restrictive criteria for additional ILI-related search terms to be included [14]. By September 2009 the historical GFT model was replaced with retrospective estimates from the revised algorithm. Currently, the updated GFT model provides real-time estimates of influenza intensity at three geographic scales in the US: national, state and select local cities, as well as estimates for many countries worldwide [16].

The original and updated GFT models have both shown high retrospective correlation with national and regional ILI disease surveillance data [13], [14]; however, the prospective accuracy of this surveillance tool remains unclear, even though GFT estimates are used in forecasting models for influenza incidence [15], [20], [21]. We present a comparative analysis of traditional public health ILI surveillance data and GFT estimates for ten influenza seasons to assess the retrospective and prospective performances of GFT to capture season-to-season epidemic timing and magnitude.

## Methods

### Public Health ILI Surveillance and Internet Search Query Data

We compared weekly ILI and GFT data from June 1, 2003 through March 30, 2013, a period of ten influenza seasons which included a range of mild and moderately severe seasonal influenza epidemics as well as the emergence of the first influenza pandemic in over forty years. The surveillance systems were assessed at three geographical levels: entire US, Mid-Atlantic region (New Jersey, New York and Pennsylvania) and New York City.

All public health surveillance data used in the study came from systems operating prospectively on a daily or weekly basis throughout the study period [2], [25]–[27]. Nationwide and regional ILI surveillance data were compiled from the US Centers for Disease Control and Prevention (CDC) sentinel ILI-Net surveillance system, which includes sources ranging from small physician practices to large electronic syndromic surveillance networks [2]. The CDC ILI-Net system is publically available each week, typically on Friday for the previous week ending Saturday during the respiratory season (October to May), with a recognized reporting lag of 1–2 weeks [2], [13]. Local ILI data came from the New York City Department of Health and Mental Hygiene (DOHMH) emergency department (ED) syndromic surveillance system, which is collected and analyzed daily, with a reporting lag of about one day [25]–[27]. In each system, all weekly public health surveillance ILI proportions were calculated as total ILI visits divided by all visits each week.

**Figure 1:**
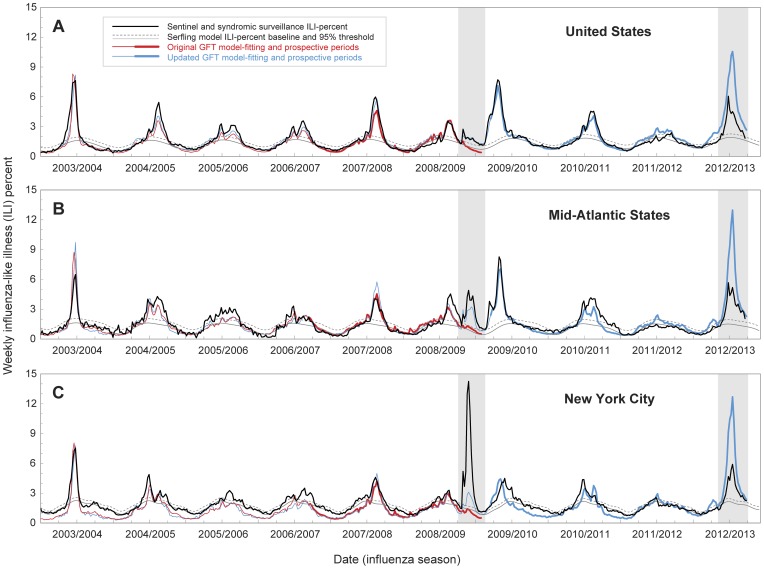
Time-series of weekly influenza-like illness (ILI) surveillance and Google Flu Trends (GFT) search query estimates, June 2003–March 2013. Observed weekly ILI proportions (black lines) are shown with Serfling model baseline (gray lines) and 95% epidemic threshold (dashed lines). The periods of the early wave of the 2009 pandemic and the 2012/2013 epidemic are shaded in grey. Sentinel ILI-Net surveillance is shown for (**A**) the United States and (**B**) Mid-Atlantic States (New Jersey, New York, Pennsylvania). Local ILI surveillance from emergency department visits is shown for (**C**) New York City. Scaled GFT internet search query estimates are shown for model-fitting periods for the original (thin red line) and updated (thin blue line) GFT models, and for the periods of prospective operation of the original (thick red line) and updated (thick blue line) GFT models. For Mid-Atlantic States the updated GFT model data represents ILI proportions only for New Jersey and New York (see Supporting Information).

Internet search query data came from the original [13] and updated GFT models [14], using weekly estimates available online [16] from both the periods of retrospective model-fitting (4 seasons for the original model and 6 seasons for the updated model) and prospective operation for both models (1 season and 4 seasons, respectively; Table 1). Finalized weekly GFT estimates were publically available each Sunday for the previous week, with a reporting lag of about one day. The original and updated GFT models used scaled measures of ILI-related searches to be directly comparable to the weighted ILI proportions from the CDC ILI-Net system [2], [13], [14], [16] (Figure 1). For additional details on data sources, see Supporting Information.

**Table 1:** Retrospective and prospective performance of original and updated Google Flu Trends (GFT) algorithm compared with national (United States), regional (Mid-Atlantic States) and local (New York City) weekly influenza-like illness (ILI) surveillance data, 2003–2013.

Time Period and Geographic Location	Original GFT model^a^	Updated GFT model^b^
	R^2^	R^2^
**National**		
Retrospective GFT model-fitting period	0.91	0.94
Prospective GFT model period	0.64	0.73
All study weeks	0.86	0.77
**Mid-Atlantic**		
Retrospective GFT model-fitting period	0.79	0.77
Prospective GFT model period	0.27	0.57
All study weeks	0.64	0.64
**New York**		
Retrospective GFT model-fitting period	0.89	0.51
Prospective GFT model period	0.03	0.77
All study weeks	0.34	0.41

Performance was evaluated by linear regression of weekly GFT estimates against weekly ILI surveillance.

a Original GFT model time periods: The retrospective query selection model-fitting period was from September 28, 2003 through March 17, 2007; the prospective GFT model validation period was from March 18, 2007 through May 17, 2008 and ongoing operation was from May 18, 2008 through Aug 1, 2009. Mid-Atlantic region states included NJ, NY and PA (13). New York comparison was based on NY state GFT estimates (16).

b Updated GFT model time periods: the retrospective query selection model-fitting period was from September 28, 2003 through September 18, 2009; The prospective operation period has run from September 19, 2009 through March 30, 2013. Mid-Atlantic region states included only NJ and NY (14). The New York level comparison was based on New York City GFT estimates (16).

### Measurement of Epidemic Timing and Intensity

All observed ILI weekly proportions were analyzed with a traditional Serfling regression approach to establish weekly expected baselines and estimate the “excess” ILI proportions attributable to influenza and identify epidemic periods ([28]–[33]; Supporting Information). The GFT system presents ILI search query estimates as a qualitative measure of influenza activity on a scale ranging from “minimal” to “intense” each week [16]; neither GFT model provided quantitative measure for detection or estimation of impact [13], [14]. For all public health surveillance and GFT estimates we assessed two epidemiological criteria to characterize influenza outbreaks: epidemic timing and intensity.

Timing was based on estimates of epidemic onset and peak week for each season and ILI surveillance system. The onset each season was defined as the first of consecutive weeks exceeding the surveillance threshold (upper limit of the 95% confidence interval of the Serfling baseline). The peak week was identified as the week with the greatest proportion of ILI visits each season or epidemic (Table 2).

**Table 2:** Comparison of seasonal and epidemic week of onset and peak weeks as measured by Google Flu Trends (GFT) and public health influenza-like illness (ILI) surveillance data at the national (United States), regional (Mid-Atlantic) and local (New York City) levels.

Time Period	National, United States			Regional, Mid-Atlantic States			Local, New York City		
	Week of Onset (Peak) ILI Surveillance	Difference in Week of Onset (Peak) Original GFT model^a^	Difference in Week of Onset (Peak) Updated GFT model^b^	Week of Onset (Peak) ILI Surveillance	Difference in Week of Onset (Peak) Original GFT model^a^	Difference in Week of Onset (Peak) Updated GFT model^b^	Week of Onset (Peak) ILI Surveillance	Difference in Week of Onset (Peak) Original GFT model^a^	Difference in Week of Onset (Peak) Updated GFT model^b^
2003/2004 season	44 (52)	+3 (−2)	+3 (0)	48 (52)	−1 (−1)	0 (0)	46 (52)	+1 (−1)	+1 (0)
2004/2005 season	51 (6)	0 (0)	0 (+1)	49 (51/6)	+1 (+2/0)	+1 (+1/+1)	47 (52)	+3 (+1)	+3 (+1)
2005/2006 season	49 (52/9)	+2 (0/0)	+2 (0/0)	48 (52/6)	+4 (+1/+3)	+4 (0/+3)	3 (6)	−2 (+3)	−3 (+1)
2006/2007 season	50 (52/7)	+1 (0/−1)	+1 (0/0)	47 (52/7)	+4 (+1/+2)	+5 (+1/+2)	47 (8)	+4 (+1)	+11 (0)
2007/2008 season	52 (7)	+1 (+1)	+3 (+1)	4 (7)	−3 (+1)	−3 (+1)	44 (7)	+9 (+1)	+9 (+1)
2008/2009 season	4 (6)	−1 (+2)	0 (+1)	4 (8)	0 (−2)	−3 (−2)	3 (7)	−2 (−1)	−2 (0)
Spring 2009 pandemic A/H1N1	17 (17)	***	0 (0)	17 (21)	***	0 (+2)	17 (21)	+3 (−1)	0 (0)
2009/2010 pandemic season	** (42)	NA	** (0)	** (43)	NA	** (+1)	34 (47)	NA	+1 (−3)
2010/2011 season	50 (5)	NA	+1 (+2)	48 (52/6)	NA	+3 (+1/+1)	46 (52)	NA	+4 (+7)
2011/2012 season	8 (11)	NA	−8 (−1)	***	NA	***	*** (52)	NA	*** (+1)
2012/2013 season	47 (52)	NA	−8 (+3)	48 (52)	NA	−9 (+3)	49 (3)	NA	−11 (0)

Week of onset was identified as the first of consecutive weeks for each system and region above its Serfling regression 95% threshold, and peaks were identified as the weeks reporting the highest percent-ILI for each season or epidemic. The public health ILI onset and peak weeks are given by surveillance week for each season. The GFT model onset and peak weeks are given relative to the corresponding season/epidemic and regional ILI surveillance weeks.

a Original GFT model time periods: The retrospective query selection model-fitting period was from September 28, 2003 through March 17, 2007; the prospective GFT model validation period was from March 18, 2007 through May 17, 2008 and ongoing operation was from May 18, 2008 through Aug 1, 2009. Mid-Atlantic region states included NJ, NY and PA (13). New York comparison was based on NY state GFT estimates (16).

b Updated GFT model time periods: the retrospective query selection model-fitting period was from September 28, 2003 through September 18, 2009; The prospective operation period has run from September 19, 2009 through March 30, 2013. Mid-Atlantic region states included only NJ and NY (14). The New York level comparisons was based on New York City GFT estimates (16).

** National and Mid-Atlantic region data remained above threshold at the beginning of the 2009/2010 pandemic season.

*** No consecutive weeks above threshold to identify onset or peak during this period.

For each data source and season we assessed epidemic intensity by determining the proportion of excess ILI for peak weeks and by summing the weekly excess ILI proportions for each epidemic period as a measure of cumulative ILI intensity for each season and epidemic. All Serfling regression confidence intervals represented the upper and lower 95% limit, calculated as the predicted non-epidemic baseline ±1.96 standard deviations [28]–[33]. We calculated the ratio of excess GFT divided by excess ILI at each geographic level for each epidemic (Table 3), with a constant ratio indicating consistent influenza monitoring by GFT for the period.

**Table 3:** Comparison of epidemic intensity during the 2009 A/H1N1 influenza pandemic and the 2012/2013 seasonal A/H3N2 epidemic as measured by Google Flu Trends (GFT) and public health influenza-like illness (ILI) surveillance at the national (United States), regional (Mid-Atlantic) and local (New York City) levels.

	Epidemic peak			Epidemic intensity as percent over baseline			Comparison GFT to ILI surveillance
Time Period and Geographic Location	ILI% at peak week			seasonal excess (95% CI)			ratio excess GFT∶ILI
	ILI surveillance	original GFT model	updated GFT model	ILI surveillance	original GFT model	updated GFT model	prospective (retrospective)
**National, United States**							
Spring 2009 pandemic A/H1N1	2.7	1.5	2.1	10.3 (6.1–14.5)	0.3 (0.1–0.6)	9.7 (5.5–13.9)	0.03 (0.94)
Autumn 2009 pandemic A/H1N1	7.7	NA	7.1	59.2 (51.8–66.5)	NA	43.8 (37.9–49.8)	0.74
2009 pandemic A/H1N1, both waves	7.7	NA	7.1	69.4 (57.9–81.0)	NA	53.5 (43.4–63.7)	0.77
2012/2013 seasonal A/H3N2	6.1	NA	10.6	27.3 (21.7–32.9)	NA	73.2 (63.7–82.6)	2.68
**Regional, Mid-Atlantic States**							
Spring 2009 pandemic A/H1N1	4.9	1.4	3.2	27.2 (21.9–32.5)	0.6 (0.03–1.1)	19.2 (15.4–23.0)	0.02 (0.71)
Autumn 2009 pandemic A/H1N1	8.3	NA	7	52.1 (42.8–61.3)	NA	40.2 (33.5–46.9)	0.77
2009 pandemic A/H1N1, both waves	8.3	NA	7.1	79.3 (64.7–93.8)	NA	59.4 (48.9–70.0)	0.75
2012/2013 seasonal A/H3N2	5.7	NA	13	34.3 (27.3–41.4)	NA	71.4 (65.9–76.8)	2.08
**Local, New York City**							
Spring 2009 pandemic A/H1N1	14.3	1.4	3.1	55.5 (52.2–58.8)	1.3 (0.4–2.1)	15.4 (10.9–19.8)	0.02 (0.28)
Autumn 2009 pandemic A/H1N1	4.5	NA	4.4	26.5 (19.0–34.0)	NA	24.3 (18.8–29.9)	0.92
2009 pandemic A/H1N1, both waves	14.3	NA	4.4	82.0 (71.2–92.8)	NA	39.7 (29.7–49.7)	0.48
2012/2013 seasonal A/H3N2	5.9	NA	12.7	26.3 (21.2–31.42)	NA	77.9 (68.2–87.5)	2.96

Epidemic intensity was measured by Serfling regression of weekly percent-ILI for public health surveillance data and GFT estimates for peak week and seasonal epidemic excess, with corresponding upper and lower 95% limit, calculated as the predicted non-epidemic baseline +1.96 standard deviations.

### Estimating Accuracy of Internet Search Query Data

To further evaluate the week-to-week accuracy and timing of GFT and potential asynchrony with traditional ILI surveillance, we calculated Pearson correlations in the national, regional and local datasets, following the original methods used in the development [13] and evaluation of GFT [14]. Original and updated GFT model estimates were assessed for the periods of retrospective model-fitting and prospective monitoring (Table 2), and for specific epidemic seasons (Table 4). We measured cross-correlations at negative and positive lags for each influenza season to identify the corresponding lead or lag with the highest correlation values between GFT and traditional ILI systems, indicating the degree of shift in the timing of the GFT trends compared to ILI surveillance.

**Table 4:** Performance of Google Flu Trends (GFT) relative to public health influenza-like illness (ILI) surveillance at the national (United States), regional (Mid-Atlantic States) and local (New York City) levels for specific epidemic and pandemic seasons.

Time Period and Geographic Location	Original GFT model	Updated GFT model
	R^2^	R^2^ ('+/− week lag, max R^2^)
**National, United States**		
Influenza seasons 2003–2009 (prior to 2009 pandemic)	0.88	0.92
2009 pandemic A/H1N1 early wave	0.91	0.84
2009/2010 pandemic A/H1N1 season	NA	0.98
2010/2011 season	NA	0.95
2011/2012 season	NA	0.88
2012/2013 season	NA	0.90
**Regional, Mid-Atlantic States**		
Influenza seasons 2003–2009 (prior to 2009 pandemic)	0.75	0.77
2009 pandemic A/H1N1 early wave	0.51	0.82
2009/2010 pandemic A/H1N1 season	NA	0.92
2010/2011 season	NA	0.83
2011/2012 season	NA	0.37
2012/2013 season	NA	0.86
**Local, New York City**		
Influenza seasons 2003–2009 (prior to 2009 pandemic)	0.87	0.84
2009 pandemic A/H1N1 early wave	0.78	0.88
2009/2010 pandemic A/H1N1 season	NA	0.51 (−3 wks, 0.89)
2010/2011 season	NA	0.74 (+1 wk, 0.80)
2011/2012 season	NA	0.80
2012/2013 season	NA	0.94

While correlations are useful to assess GFT [14], they only provide a measure of relative correspondence between ILI and internet search systems, and do not provide an indication of the nature of the relationship between the trend estimates or the observed lags. As a complementary measure, we compared the regression slope of public health ILI data with GFT estimates during retrospective model-fitting and prospective periods, and for specific seasons. For further details, see Supporting Information.

## Results

During the study period, June 2003 to March 2013, over 4.5 million ILI visits out of 230 million total outpatient sentinel physician visits were reported nationwide to the CDC ILI-Net surveillance network, of which 16.5% were from the Mid-Atlantic surveillance region. In New York City, over 780,000 ILI and 38 million total ED visits were recorded in the DOHMH syndromic surveillance system, with coverage increasing from 88% of all ED visits that occurred citywide during 2003/2004 to >95% of all visits since 2008. The weekly proportion of ILI visits and GFT estimates showed similar seasonal and epidemic patterns across the three regional scales, though with notable differences between retrospective and prospective periods (Figure 1; Table 1). Specifically, during prospective use the original GFT algorithm severely underestimated the early 2009 pandemic wave (shaded 2009 period, Figure 1), and the updated GFT model greatly exaggerated the intensity of the 2012/2013 influenza season (shaded 2012/2013 period, Figure 1).

### Original GFT Model, 2003–2009 Prior to the Pandemic

**Figure 2:**
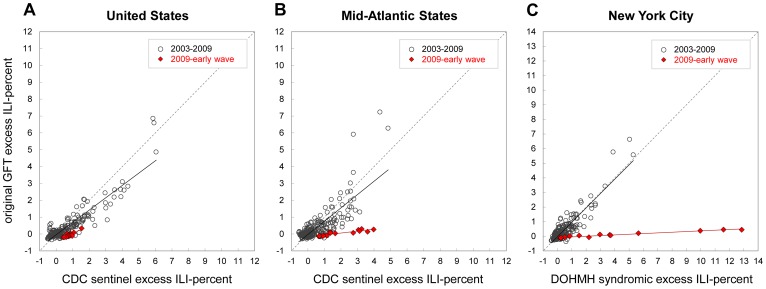
Scatter plots of weekly excess influenza-like illness (ILI) visit proportions against original Google Flu Trends (GFT) model search query estimates, 2003–2009. Weekly excess percent-ILI is calculated as Serfling estimates subtracted from observed proportions. Plots show original GFT model estimates compared with weighted CDC ILI-Net data for (**A**) the United States, and (**B**) Mid-Atlantic Census Region States (New Jersey, New York, Pennsylvania), and local ILI surveillance from emergency department visits for (**C**) New York City. Plots are shown for pre-pandemic influenza seasons, June 1, 2003 to April 25, 2009 (grey circles) and the early wave of the A/H1N1 pandemic, April 26 to August 1, 2009 (red diamonds). Lines representing equivalent axes for X = Y are shown (grey dashed line). Regression lines are shown for seasonal influenza 2003–2009 (black line) and the early 2009 wave of the pandemic (red line).

Historical estimates from the original GFT model were based on the model-fitting period from September 28, 2003 to March 17, 2007; the system was evaluated during March 18, 2007 to May 11, 2008, and has run prospectively since then. The week-to-week GFT estimates during the model-fitting period were highly correlated with ILI surveillance data at the national (R^2^ = 0.91), regional (Mid-Atlantic, R^2^ = 0.79) and state/local level (New York, R^2^ = 0.89; Table 1). Similarly, GFT estimates were highly correlated with CDC ILI surveillance at the national and regional levels during the validation period [13], and remained high through the period of prospective use prior to the emergence of the 2009 A/H1N1 pandemic, from May 12, 2008 to March 28, 2009 (R^2^≥0.75; Table 4). Seasonal and epidemic onset and peak weeks varied considerably during the period (Table 2). Estimation of excess ILI visits and GFT search query fractions were also well correlated on a week to week basis during this period (Supporting Tables; Figure 2).

### Original GFT Model during the First Wave of the 2009 Pandemic

In late-April 2009, detection of novel A/H1N1 influenza in an outbreak in Queens, New York, was immediately followed by a spike in ILI surveillance data across much of the nation during the week ending May 2, 2009 [2]. Mid-Atlantic States and New York City experienced a substantial spring pandemic wave (Figure 1B,C), unlike many other regions of the US [2]. Despite recognized pandemic activity, the national GFT estimates were below baseline ILI levels for May–August 2009, indicating no excess impact (red line, shaded 2009 period, Figure 1A). The correlations between the surveillance ILI and GFT estimates, however, were very high during this period at the US level for observed (R^2^ = 0.91) as well as estimated excess values (R^2^ = 0.81; Figure 2A). At the Mid-Atlantic level, correlations were lower for observed (R^2^ = 0.51), but still high for estimated excess values (R^2^ = 0.80), while the slope of the linear relationship between the two surveillance systems was near zero (slope = 0.11), indicating that there was little or no excess ILI estimated by GFT (Figure 2B). The discrepancy at the Mid-Atlantic level was exacerbated for New York City, where the pandemic impact was greater than any other epidemic that decade, while the original GFT estimates remained near expected baseline levels for the entire period (R^2^ = 0.78). Accordingly, the slope of the GFT regression against ILI was near zero (slope = 0.05), indicating that GFT data did not accurately measure the intensity of the pandemic (Figure 2C). Taken together, the original GFT model missed the spring 2009 pandemic wave at all levels (Figure 1), providing incidence estimates 30–40 fold lower than those based on ILI surveillance (Table 3).

### Updated GFT Model, Retrospective Period 2003–2009

The original and updated GFT estimates appeared very similar during the pre-pandemic period 2003–2009, but diverged considerably by May 2009 (red and blue lines, Figure 1). Like the original GFT model, the updated GFT estimates during the model-fitting period were highly correlated with CDC ILI surveillance at the national and regional levels (R^2^≥0.77, Table 1). In contrast for New York City, the updated GFT estimates were less well correlated with local ILI syndromic surveillance data during this period (R^2^ = 0.51, Table 1). Of particular interest is the retrospective characterization of the 2009 pandemic by the updated GFT algorithm, which tracked the spring wave very well at the national level, but underestimated the magnitude at the regional level by nearly 30%, and at the New York City level by 70% (Figure 1; Table 3).

### Updated GFT Model Ability to Track the Fall 2009 Pandemic

In September 2009, the updated GFT algorithm began running prospectively, providing estimates that tracked CDC ILI surveillance data well for the remainder of 2009, a period in which most pandemic A/H1N1 infections occurred. Updated GFT estimates were highly correlated with ILI surveillance at the national (R^2^ = 0.98), and regional (R^2^ = 0.92) levels (Figure 1A–B; Table 4). Mid-Atlantic ILI surveillance, however, demonstrated two peaks, consistent with different timing of pandemic waves in states within the region (Figure 1B). For New York City, the updated GFT estimates and ILI surveillance were less well correlated when measured directly (R^2^ = 0.51), though highly correlated when lagged by three weeks (R^2^ = 0.89), showing the updated GFT model estimates for the fall 2009 pandemic wave to increase and peak 3 weeks earlier than ILI surveillance (Figure 1C; Table 4). Overall, GFT underestimated the cumulative ILI incidence of the main pandemic period, May–December 2009, by 52% for New York City (25% for the broader region), with non-overlapping confidence intervals between the GFT and ILI surveillance systems (Table 3).

### Updated GFT Model Performance during 2010–2012

Correlations between the updated GFT model and ILI data during the first two years of prospective post-pandemic surveillance were high at the national level during the 2010/2011 (R^2^ = 0.95) and 2011/2012 (R^2^ = 0.88) seasons (Table 4). At the regional level, there was high correlation in 2010/2011 (R^2^ = 0.83) with a slight underestimation of incidence, and low correlation in 2011/2012 (R^2^ = 0.37) with a slight overestimation of ILI incidence (Figure 1B). At the New York City level, updated GFT estimates for 2010/2011 were reasonably well correlated with observed ILI (R^2^ = 0.74), though with ILI surveillance increasing and peaking earlier (Figure 1C), and showing an improved lagged correlation (R^2^ = 0.80, lagged 1 week; Table 4).

### Updated GFT Model Performance during the 2012/2013 Season

**Figure 3:**
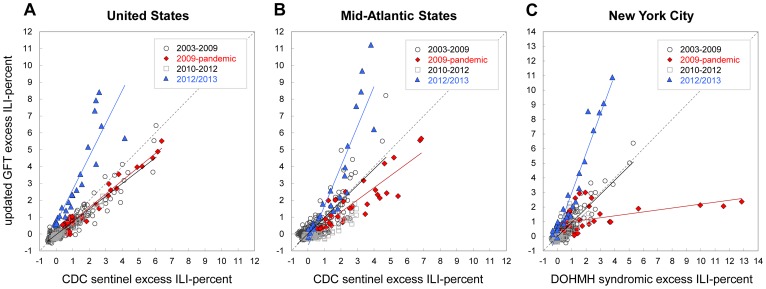
Scatter plots of weekly excess influenza-like illness (ILI) visit proportions against updated Google Flu Trends (GFT) model search query estimates, 2003–2013. Weekly excess percent-ILI is calculated as Serfling estimates subtracted from observed proportions. Plots show updated GFT model estimates compared with weighted CDC ILI-Net data for (**A**) the United States, and (**B**) Mid-Atlantic HHS-2 Region States (New Jersey, New York), and local ILI surveillance from emergency department ILI visit data for (**C**) New York City. Plots are shown for weeks June 1, 2003 to April 25, 2009 (grey circles), April 26 to January 2, 2010 (red diamonds), January 3, 2010 to Oct 6, 2012 (grey squares), and October 7, 2012 to March 30, 2013 (blue triangles). Lines representing equivalent axes for X = Y are shown (grey dashed line). Regression lines are shown for the 2003/2004–2008/2009 seasons (black line), 2009 pandemic (red line), 2010/2011–2010/2012 seasons (grey solid line) and the 2012/2013 season (blue line).

For the relatively early and moderately severe 2012/2013 epidemic season, observed GFT estimates greatly overestimated the initial onset week and magnitude of the outbreak at all three geographical levels (Figure 1; Table 2). The correlations between the updated GFT model estimates and ILI surveillance, however, were very high at all levels (R^2^≥0.86, Table 4). GFT model estimates of epidemic intensity were far greater than ILI surveillance data at the national (268%), regional (208%) and local (296%) levels (Table 3). Accordingly, the slopes of the weekly regression of ILI surveillance against GFT estimates during 2012/2013 (United States, slope = 1.91; Mid-Atlantic, slope = 2.29; New York City, slope = 2.63) were far greater than those for other epidemic and pandemic seasons (Figure 3), and substantially different from a slope of 1 (p<0.05).

## Discussion

Following Google's development of GFT in 2008, and the considerable excitement generated by the original publication and release of the online tool [12], [13], [16], concerns were raised regarding the tenuous relationship between internet searches and the presentation of illness to clinical or emergency medical providers [17]. We used clinical ILI surveillance data at local, regional and national scales as a proposed “ground truth” to test the ability of GFT to perform as a timely and accurate surveillance system in the US. We identified substantial errors in GFT estimates of influenza timing and intensity in the face of pandemic and seasonal outbreaks, including prospectively missing the early wave of the 2009 pandemic and overestimating the impact of the 2012/2013 epidemic. Although we are not the first to report issues in GFT estimates for seasonal and pandemic influenza [22], our study is the first to carefully quantify the performance of this innovative system over a full decade of influenza activity and across three geographical scales.

The 2009 A/H1N1 pandemic is a particularly important case study to test the performance of GFT, with its unusual signature pandemic features of out-of-season activity in the spring of 2009, atypical (young) age pattern of cases, recurring waves and substantial geographic heterogeneity [34]–[38]. Immediately following the spread of the pandemic virus in the US, public health officials and electronic surveillance networks found that local and state level surveillance data did not correspond with estimates provided by the original GFT model, particularly in some urban areas and harder hit regions of the Northeastern and Midwestern US [18], [39]. Clearly, the original GFT algorithm was not able to track sentinel ILI patterns that deviated from the influenza seasons that occurred during the model-fitting period. Even after the GFT algorithm was revised in September 2009, we have shown that the retrospective estimates for the spring 2009 pandemic wave were still not in agreement with regional and local surveillance. Further, the updated GFT model that has been used prospectively failed to accurately capture the autumn 2009 pandemic wave in New York City, presenting the outbreak three weeks before it actually occurred. This assessment echoes earlier concerns regarding the timeliness and accuracy of internet search data for public health monitoring at the local level [17] and during the early wave of the 2009 pandemic [18]. To have missed the early wave of the 2009 pandemic is a serious shortcoming of a surveillance system – as these are times when accurate data are most critically needed for pandemic preparedness and response purposes.

Although the GFT system provided relatively accurate estimates during post-pandemic years which were characterized by mild influenza activity, it overestimated the 2012/2013 epidemic by 2–3 fold relative to traditional ILI surveillance systems, across national, regional and local geographical levels in the US (see also [22]). While the intensity of the 2012/2013 influenza season was roughly comparable to the 2003 A/H3N2-Fujian epidemic as measured by traditional surveillance and assessed by CDC as “moderately severe” [2], the 2012/2013 season was scored by the GFT tool as by far the most severe epidemic in over a decade.

A limitation of our study is its focus on US systems. Many international syndromic, physician consultation, laboratory and internet survey surveillance systems provide rapid, detailed and accurate influenza-related surveillance [3]–[5], [40]–[48]. These systems allowed for development of GFT search query algorithms which were trained to mimic the specific regional influenza-related patterns [16]. While international GFT search query estimates are publically available earlier than many government run surveillance systems, it is important to note that public health data typically undergo monitoring for data quality and investigation prior to public release. It is also important to note that GFT has been set up where robust surveillance systems already exist, providing ILI search query data for populations that are already under surveillance.

An additional limitation of our study is the imperfect nature of our assumed “ground truth” surveillance. Our study sought to assess the ability of GFT to estimate physician consultation and syndromic ILI surveillance patterns, not necessarily the true incidence of influenza infection and illness. We recognize that physician sentinel and syndromic data can be biased, particularly during periods of heightened public health concern. This has been well described in a study of online survey data and health-seeking behavior during the two waves of the 2009 pandemic in England [48]. This recognized bias highlights the need for multiple sources of surveillance information in the community.

In a previous evaluation of GFT, the authors and engineers at Google and the US CDC concluded that their original GFT model had “performed well prior to and during” the 2009 pandemic, when assessed as simple correlations at national and regional levels [14]. Regarding this measure of performance, however, we found the use of simple correlation to be inadequate, as values greater than 0.90 often occurred during periods when critical metrics such as peak magnitude and cumulative ILI revealed that the GFT models were actually greatly under- or over-estimating influenza activity. Our study demonstrates that simple correlation measures can mischaracterize the performances of a novel surveillance system, and instead we recommend the use of additional and alternative metrics based on estimates of onset and peak timing and cumulative intensity of influenza epidemics.

Because the search algorithm and resulting query terms that were used to define the original and updated GFT models remain undisclosed, [13], [14], it is difficult to identify the reasons for the suboptimal performance of the system and make recommendations for improvement. Concerns were raised early-on that the data-mining nature of GFT might over-fit the historical data and introduce bias in prospective use [17]. After the original GFT model missed the spring 2009 pandemic wave – an outbreak with different timing and characteristics than the outbreaks present in the retrospective model-fitting period – the GFT algorithm was modified, potentially addressing the possible over-fitting issue. The revised GFT model, however, appeared to be susceptible to bias in the opposite direction, possibly due to changes in health information searching and care seeking behavior driven by the media. Further, important epidemiologic information such as patient age, location, illness complaint or clinical presentation remain un-available in GFT (an adult person could be performing a search on behalf of a sick minor in another state). In contrast, public health information systems are less prone to such biases, as they collect demographic and geographic data as well as additional health outcomes, which can be used to investigate atypical signals.

Ultimately, public health actions are taken locally. As such, the accuracy and timeliness of *local* disease surveillance systems are critical; as is the utility of the information in supporting decisions. The additional detail in local syndromic ILI surveillance data, and its direct link to individuals seeking care, facilitates public health action. Computerized surveillance, such as the New York City syndromic chief complaint ED system, can accurately capture the impact of influenza activity [25], [26]. In the present study, we have shown that these systems are more accurate than, yet equally timely as the GFT tool, which indicates the need for further research and support for computerized local disease surveillance systems.

We believe there is a place for internet search query monitoring in disease surveillance, and for continued research and development in this area [13]–[21], [49]–[58]. For now, in the US CDC's national and regional ILI surveillance data remain the “ground truth” source of influenza activity at national and regional levels, but timeliness, detail and coverage remain issues. Thus, we believe there is a broader need for electronic clinically-based disease surveillance at the local level, similar to the ED system in place in New York City [25]–[27], and for collaborative and distributed networks connecting these systems for research and practice [39], [58]–[60]. Careful evaluation of the strengths and limitations of GFT and other innovative surveillance tools should be expanded to encompass a range of developed and developing country settings, following the approach proposed here, in order to improve local, regional and global outbreak surveillance methods and inform public health responses. The way forward using high volume search query data such as GFT may be through integration of near-real time electronic public health surveillance data, improved computational methods and disease modeling – creating systems that are more transparent and collaborative, as well as more rigorous and accurate, so as to ultimately make them of greater utility for public health decision making.

## Supporting Information

Figure S1 **National level influenza season observed and model baseline data, 2003–2013.**
(PDF)

Figure S2 **Comparison of national level influenza-like illness (ILI) surveillance and Google Flu Trends (GFT) original and updated models.**
(PDF)

Figure S3 **Mid-Atlantic state seasonal observed and model baseline, 2003–2013.**
(PDF)

Figure S4 **Comparison of Mid-Atlantic state influenza-like illness (ILI) surveillance and Google Flu Trends (GFT) original and updated models.**
(PDF)

Figure S5 **New York influenza season observed and model baseline data, 2003–2013.**
(PDF)

Figure S6 **Comparison of New York State and New York City Google Flu Trends (GFT) updated models.**
(PDF)

Figure S7 **Comparison of New York City emergency department (ED) influenza-like illness (ILI) syndrome surveillance and New York City and State Google Flu Trends (GFT) original and updated models.**
(PDF)

Table S1 **Influenza epidemic season intensity, national level in the United States, 2003–2013.**
(PDF)

Table S2 **Google Flu Trends (GFT) model correlation, national level in United States, 2003–2013.**
(PDF)

Table S3 **Influenza season epidemic intensity in Mid-Atlantic States, 2003–2013.**
(PDF)

Table S4 **Google Flu Trends (GFT) model correlation, Mid-Atlantic States, 2003–2013.**
(PDF)

Table S5 **Influenza season epidemic intensity in New York, 2003–2013.**
(PDF)

Table S6 **Google Flu Trends (GFT) model correlation, New York, 2003–2013.**
(PDF)

Text S1 **Technical appendix: Supplementary methods and results.**
(PDF)
