# Supplementary figures and images for: Reassessing Google Flu Trends Data for Detection of Seasonal and Pandemic Influenza: A Comparative Epidemiological Study at Three Geographic Scales
Source: PLoS Comput Biol. 2013 Oct 17;9(10):e1003256. doi: 10.1371/journal.pcbi.1003256 (PMC3798275; doi:10.1371/journal.pcbi.1003256)

**Figure S1 – National level influenza season observed and model baseline data, 2003-2013**

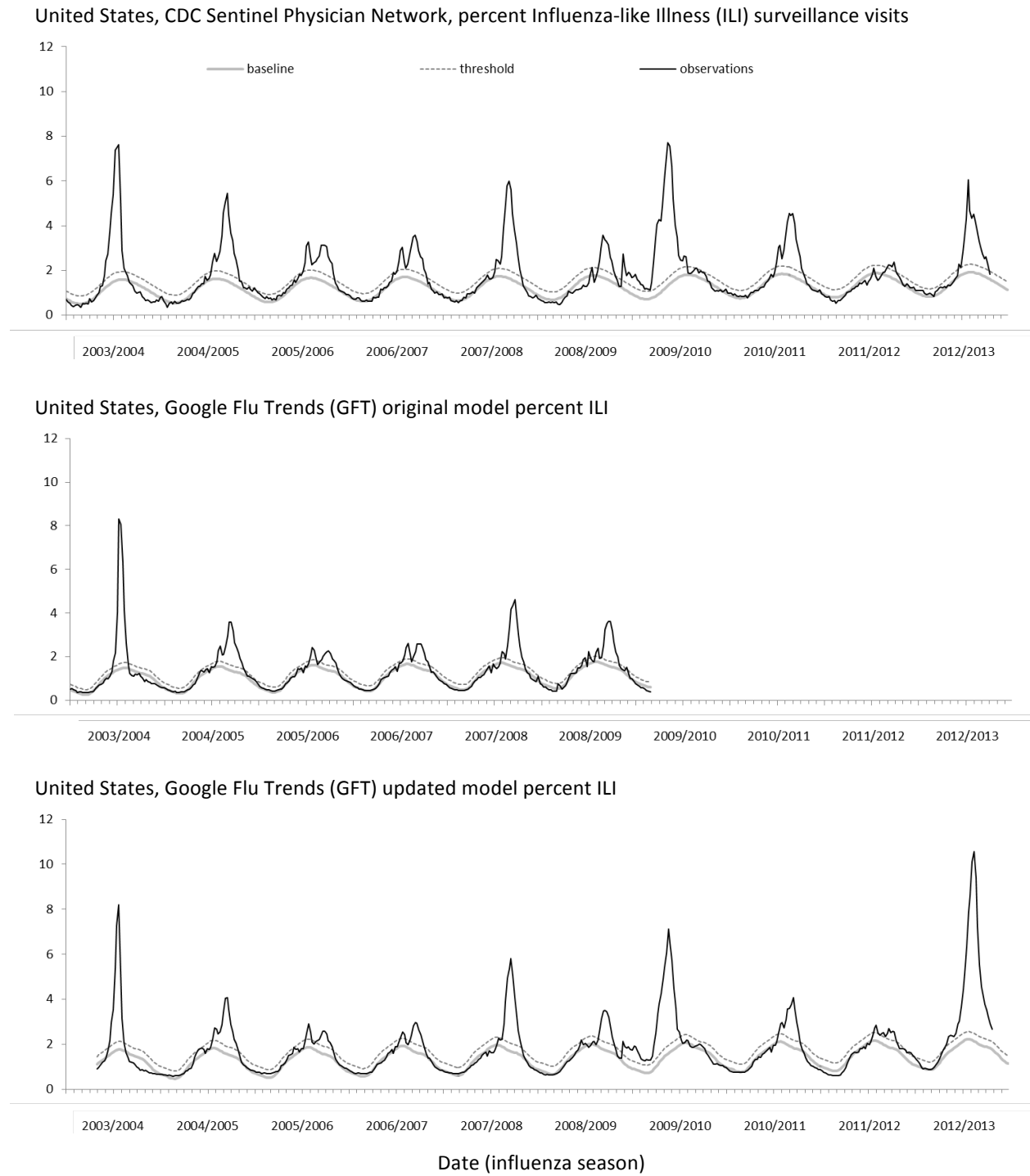

Supplement: Figure S1 — National level influenza season observed and model baseline data, 2003–2013. (PDF) [file pcbi.1003256.s001.pdf]

**Figure S5 – New York influenza season observed and model baseline data, 2003-2013**

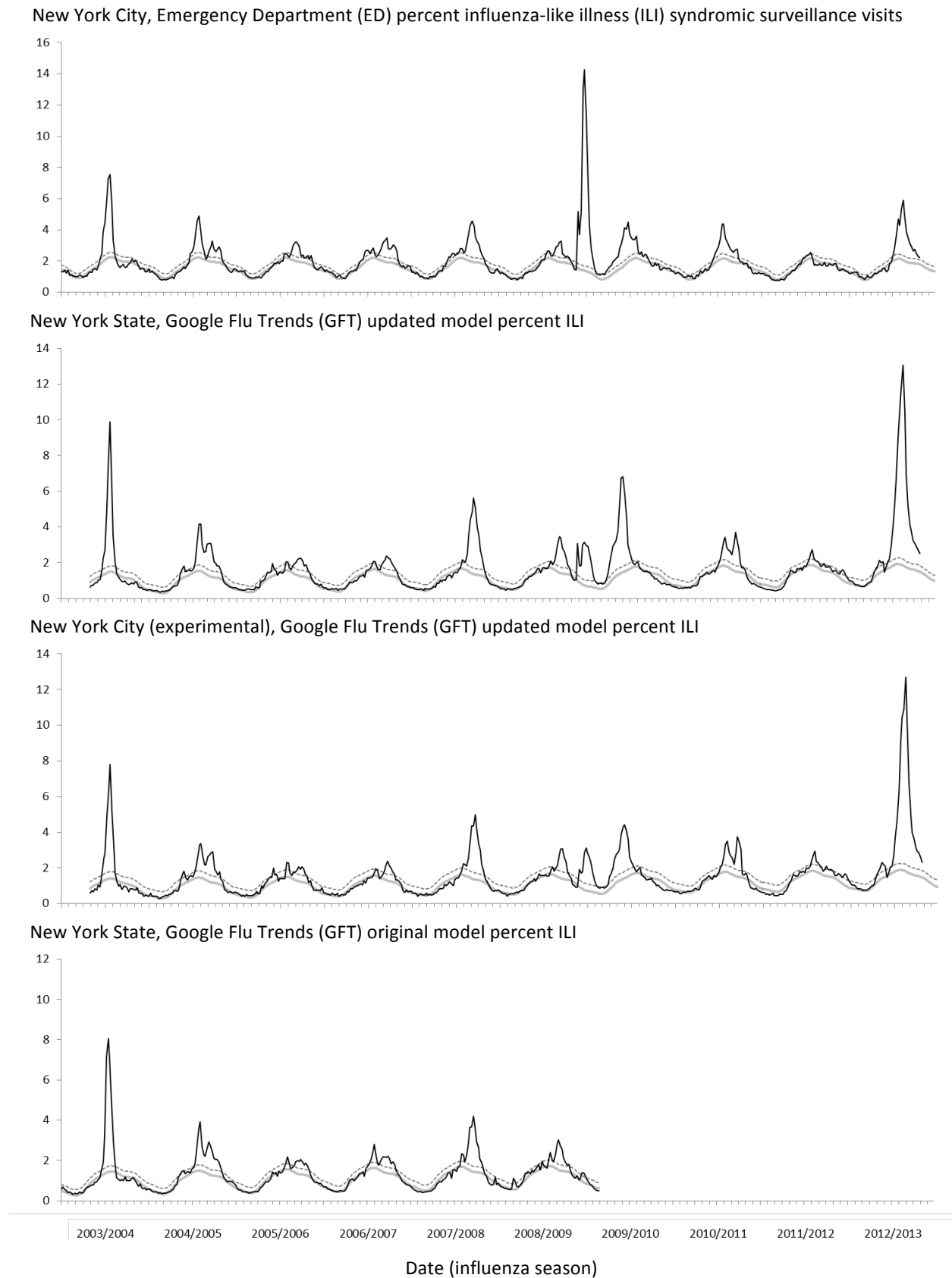

Supplement: Figure S5 — New York influenza season observed and model baseline data, 2003–2013. (PDF) [file pcbi.1003256.s005.pdf]

Figure S6 – Comparison of New York State and New York City Google Flu Trends (GFT) updated models

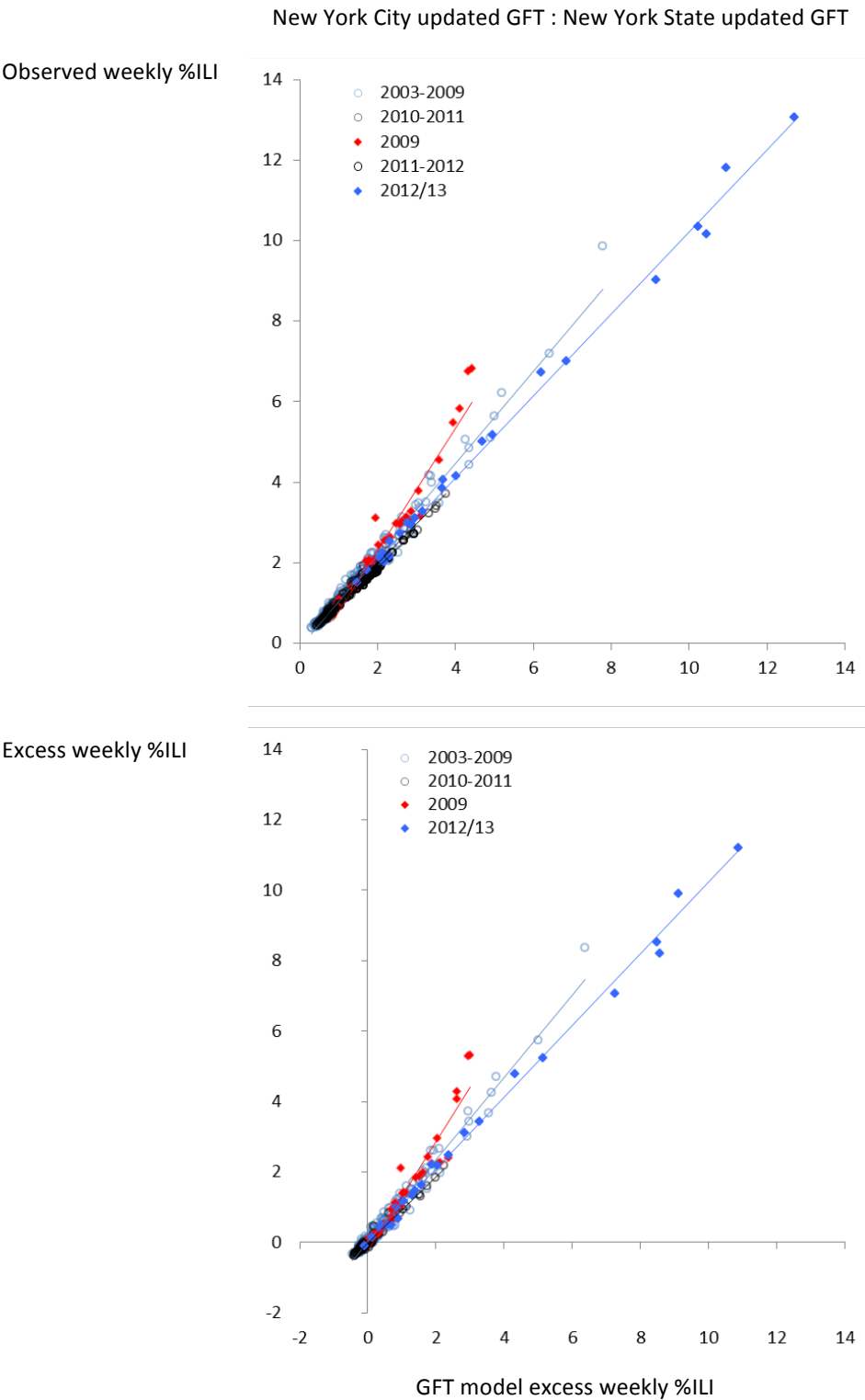

Supplement: Figure S6 — Comparison of New York State and New York City Google Flu Trends (GFT) updated models. (PDF) [file pcbi.1003256.s006.pdf]
