# Supplementary material for: Reassessing Google Flu Trends Data for Detection of Seasonal and Pandemic Influenza: A Comparative Epidemiological Study at Three Geographic Scales
Source: PLoS Comput Biol. 2013 Oct 17;9(10):e1003256. doi: 10.1371/journal.pcbi.1003256 (PMC3798275; doi:10.1371/journal.pcbi.1003256)

**Figure S2 – Comparison of national level influenza-like illness (ILI) surveillance and Google Flu Trends (GFT) original and updated models**

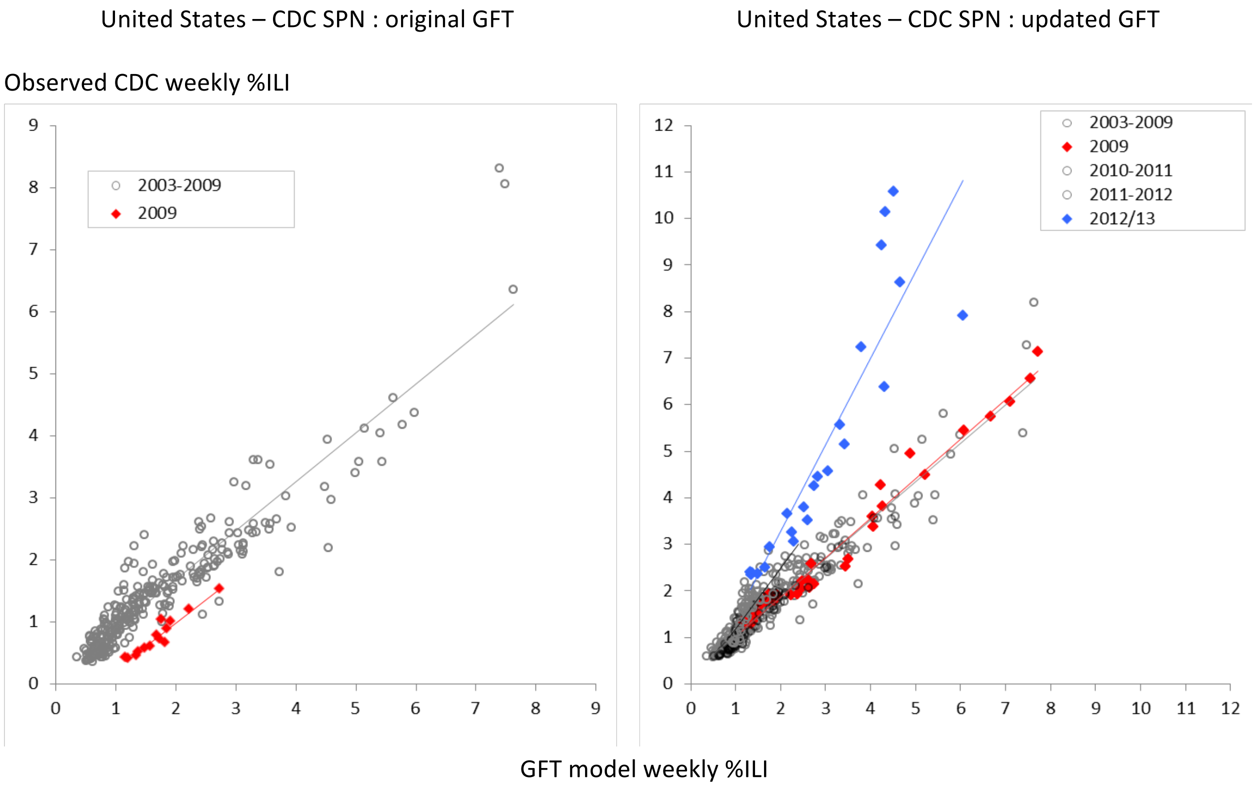

Supplement: Figure S2 — Comparison of national level influenza-like illness (ILI) surveillance and Google Flu Trends (GFT) original and updated models. (PDF) [file pcbi.1003256.s002.pdf]
