# Supplementary material for: Reassessing Google Flu Trends Data for Detection of Seasonal and Pandemic Influenza: A Comparative Epidemiological Study at Three Geographic Scales
Source: PLoS Comput Biol. 2013 Oct 17;9(10):e1003256. doi: 10.1371/journal.pcbi.1003256 (PMC3798275; doi:10.1371/journal.pcbi.1003256)

Figure S3 – Mid-Atlantic State seasonal observed and model baseline, 2003-2013

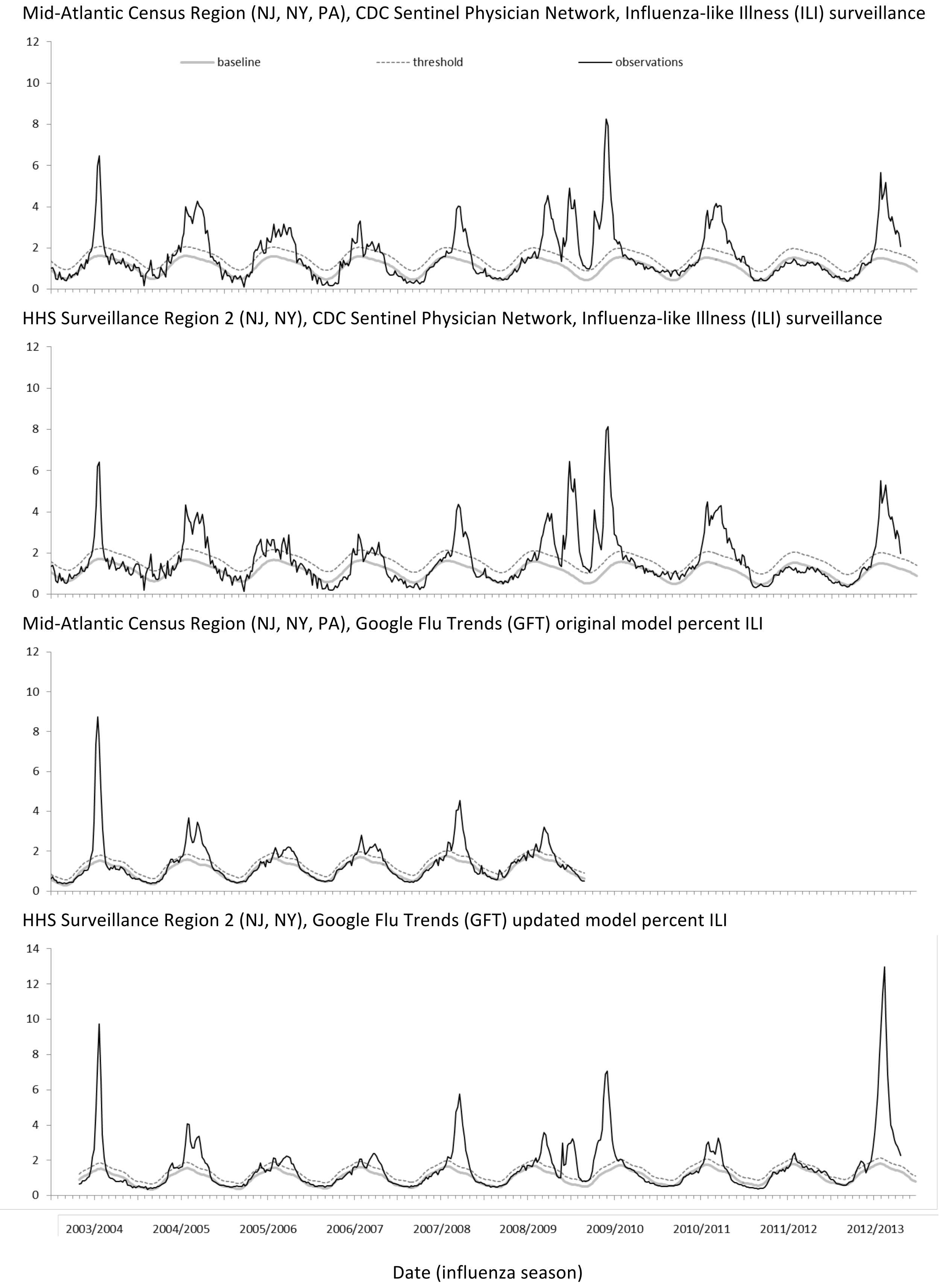

Supplement: Figure S3 — Mid-Atlantic state seasonal observed and model baseline, 2003–2013. (PDF) [file pcbi.1003256.s003.pdf]
