# Supplementary material for: Reassessing Google Flu Trends Data for Detection of Seasonal and Pandemic Influenza: A Comparative Epidemiological Study at Three Geographic Scales
Source: PLoS Comput Biol. 2013 Oct 17;9(10):e1003256. doi: 10.1371/journal.pcbi.1003256 (PMC3798275; doi:10.1371/journal.pcbi.1003256)
Supplement: Figure S7 — Comparison of New York City emergency department (ED) influenza-like illness (ILI) syndrome surveillance and New York City and State Google Flu Trends (GFT) original and updated models. (PDF) [file pcbi.1003256.s007.pdf]

Figure S7 – Comparison of New York City emergency department (ED) influenza-like illness (ILI) syndrome surveillance and New York State Google Flu Trends (GFT) original and updated models

NYC ED ILI : original NY GFT ILI | NYC ED ILI : updated NY GFT ILI | NYC ED ILI : updated NYC GFT ILI

Observed weekly (%ILI)

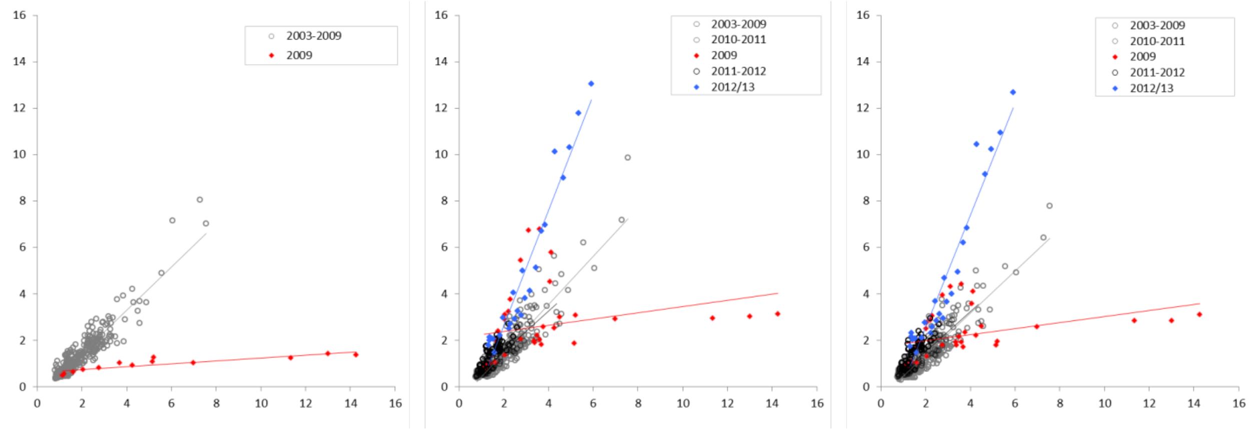

GFT model weekly %ILI
