# Supplementary material for: Reassessing Google Flu Trends Data for Detection of Seasonal and Pandemic Influenza: A Comparative Epidemiological Study at Three Geographic Scales
Source: PLoS Comput Biol. 2013 Oct 17;9(10):e1003256. doi: 10.1371/journal.pcbi.1003256 (PMC3798275; doi:10.1371/journal.pcbi.1003256)
Supplement: Table S1 — Influenza epidemic season intensity, national level in the United States, 2003–2013. (PDF) [file pcbi.1003256.s008.pdf]

Table S1 – Influenza epidemic season intensity, national level in the United States, 2003-2013

United States, CDC Sentinel Physician Network, Influenza-like Illness (ILI) Surveillance

Epidemic Season	Epidemic period weeks	excess	lower 95%	upper 95%	peak
2003/2004	12	35.66	31.46	39.86	7.63
2004/2005	15	27.62	22.37	32.87	5.44
2005/2006	18	19.49	13.19	25.79	3.28
2006/2007	16	17.72	12.12	23.32	3.58
2007/2008	14	29.75	24.85	34.65	5.98
2008/2009	9	11.33	8.18	14.48	3.57
spring-2009	12	10.25	6.05	14.45	2.74
2009/2010	21	59.20	51.85	66.55	7.72
2010/2011	16	24.46	18.86	30.06	4.55
2011/2012	5	2.68	0.93	4.43	2.38
2012/2013	11	24.14	20.29	27.99	6.09

United States, Google Flu Trends (GFT) original model

Epidemic Season	Epidemic period weeks	excess	lower 95%	upper 95%	peak
2003/2004	9	26.20	24.04	28.36	8.30
2004/2005	16	16.69	12.85	20.53	3.57
2005/2006	15	8.30	4.70	11.90	2.43
2006/2007	14	9.71	6.35	13.07	2.61
2007/2008	13	18.20	15.08	21.32	4.61
2008/2009	10	12.78	10.38	15.18	3.62
spring-2009	1	0.34	0.10	0.58	1.53
2009/2010	NA	NA	NA	NA	NA
2010/2011	NA	NA	NA	NA	NA
2011/2012	NA	NA	NA	NA	NA
2012/2013	NA	NA	NA	NA	NA

United States, Google Flu Trends (GFT) updated model

Epidemic Season	Epidemic period weeks	excess	lower 95%	upper 95%	peak
2003/2004	8	24.35	21.55	27.15	8.20
2004/2005	15	17.98	12.73	23.23	2.72
2005/2006	15	9.82	4.57	15.07	2.92
2006/2007	10	7.73	4.23	11.23	2.97
2007/2008	10	23.21	19.71	26.71	5.81
2008/2009	9	10.95	7.80	14.10	3.50
spring-2009	12	9.70	5.50	13.90	2.13
2009/2010	17	43.82	37.87	49.77	7.11
2010/2011	12	14.87	10.67	19.07	4.08
2011/2012	5	3.45	1.70	5.20	2.86
2012/2013	22	66.30	58.60	74.00	10.56
