# Supplementary material for: Reassessing Google Flu Trends Data for Detection of Seasonal and Pandemic Influenza: A Comparative Epidemiological Study at Three Geographic Scales
Source: PLoS Comput Biol. 2013 Oct 17;9(10):e1003256. doi: 10.1371/journal.pcbi.1003256 (PMC3798275; doi:10.1371/journal.pcbi.1003256)
Supplement: Table S3 — Influenza season epidemic intensity in Mid-Atlantic States, 2003–2013. (PDF) [file pcbi.1003256.s010.pdf]

Table S3 – Influenza season epidemic intensity in Mid-Atlantic States, 2003-2013

Mid-Atlantic Census Region (NJ, NY, PA), CDC Sentinel Physician Network, Influenza-like Illness (ILI) Surveillance

Epidemic Season	weeks	excess	Epidemic period lower 95%	upper 95%	peak
2003/2004	12	18.84	13.56	24.12	6.48
2004/2005	15	30.56	23.96	37.16	4.01
2005/2006	12	13.98	8.70	19.26	3.16
2006/2007	10	6.11	1.71	10.51	3.32
2007/2008	14	17.97	11.81	24.13	4.04
2008/2009	9	19.12	15.16	23.08	4.54
spring-2009	12	27.18	21.90	32.46	4.90
2009/2010	21	52.07	42.83	61.31	8.27
2010/2011	15	29.58	22.98	36.18	4.15
2011/2012	5	-0.23	-2.43	1.97	1.47
2012/2013	11	27.43	22.59	32.27	5.74

HHS Surveillance Region 2 (NJ, NY), CDC Sentinel Physician Network, Influenza-like Illness (ILI) Surveillance

Epidemic Season	weeks	excess	Epidemic period lower 95%	upper 95%	peak
2003/2004	12	16.92	10.80	23.04	6.43
2004/2005	15	27.07	19.42	34.72	4.34
2005/2006	12	5.67	-0.45	11.79	2.90
2006/2007	10	6.23	1.13	11.33	2.91
2007/2008	14	20.06	12.92	27.20	4.38
2008/2009	9	16.96	12.37	21.55	3.95
spring-2009	12	35.76	29.64	41.88	6.45
2009/2010	21	52.13	41.42	62.84	8.12
2010/2011	15	33.79	26.14	41.44	4.48
2011/2012	5	-0.27	-2.82	2.28	1.35
2012/2013	11	29.19	23.58	34.80	5.62

Mid-Atlantic Census Region (NJ, NY, PA), Google Flu Trends (GFT) original model

Epidemic Season	weeks	excess	Epidemic period lower 95%	upper 95%	peak
2003/2004	9	28.16	25.73	30.59	8.73
2004/2005	17	19.73	15.14	24.32	3.67
2005/2006	11	6.44	3.47	9.41	2.21
2006/2007	10	6.51	3.81	9.21	2.80
2007/2008	13	18.46	14.95	21.97	4.53
2008/2009	9	8.53	6.10	10.96	3.19
spring-2009	2	0.57	0.03	1.11	1.35
2009/2010	NA	NA	NA	NA	NA
2010/2011	NA	NA	NA	NA	NA
2011/2012	NA	NA	NA	NA	NA
2012/2013	NA	NA	NA	NA	NA

HHS Surveillance Region 2 (NJ, NY), Google Flu Trends (GFT) updated model

Epidemic Season	weeks	excess	Epidemic period lower 95%	upper 95%	peak
2003/2004	12	26.52	22.68	30.36	9.73
2004/2005	15	21.62	16.82	26.42	4.06
2005/2006	12	7.56	3.72	11.40	2.23
2006/2007	10	7.26	4.06	10.46	2.40
2007/2008	14	27.05	22.57	31.53	5.75
2008/2009	9	11.52	8.64	14.40	3.59
spring-2009	12	19.21	15.37	23.05	3.24
2009/2010	21	40.23	33.51	46.95	7.05
2010/2011	15	12.87	8.07	17.67	3.25
2011/2012	5	0.69	-0.91	2.29	2.41
2012/2013	12	64.79	60.95	68.63	12.97
