# Supplementary material for: Reassessing Google Flu Trends Data for Detection of Seasonal and Pandemic Influenza: A Comparative Epidemiological Study at Three Geographic Scales
Source: PLoS Comput Biol. 2013 Oct 17;9(10):e1003256. doi: 10.1371/journal.pcbi.1003256 (PMC3798275; doi:10.1371/journal.pcbi.1003256)
Supplement: Table S4 — Google Flu Trends (GFT) model correlation, Mid-Atlantic States, 2003–2013. (PDF) [file pcbi.1003256.s011.pdf]

Table S4 – Google Flu Trends (GFT) model correlation, Mid-Atlantic States, 2003-2013

Mid-Atlantic States, CDC Sentinel Physician Network, Influenza-like Illness (ILI) Surveillance – original GFT model data represent the Mid-Atlantic Surveillance Census Region (NJ, NY, PA) and the updated GFT model represents Health and Human Services Surveillance Region 2 (NJ, NY)

	original GFT		updated GFT	
Time Period	Rsq (observed)	Rsq (excess)	Rsq (observed)	Rsq (excess)
Retrospective GFT model training periods	0.79	0.68	0.77	0.72
Prospective GFT model surveillance	0.27	0.22	0.58	0.53
All study weeks	0.64	0.49	0.65	0.59

	original GFT		updated GFT	
Time Period	Rsq (observed)	Rsq (excess)	Rsq (observed)	Rsq (excess)
Influenza seasons 2003-2009 (pre-pandemic)	0.75	0.63	0.77	0.68
March 29, 2009 - January 30, 2010	0.20	0.79	0.77	0.82
- pandemic A/H1N1-2009 spring wave	0.51	0.80	0.82	0.82
- pandemic A/H1N1-2009 fall wave	NA	NA	0.88	0.88
Influenza seasons 2010/2011-2011/2012	NA	NA	0.78	0.72
Influenza season 2012/2013	NA	NA	0.91	0.90

	original GFT		updated GFT	
Year (June-May)	Rsq (observed)	Rsq (excess)	Rsq (observed)	Rsq (excess)
2003-2004	0.95	0.95	0.93	0.92
2004-2005	0.89	0.82	0.78	0.67
2005-2006	0.88	0.44	0.68	0.13
2006-2007	0.79	0.40	0.79	0.45
2007-2008	0.95	0.91	0.92	0.89
2008-2009	0.27	0.22	0.73	0.83
2009-2010	NA	NA	0.92	0.93
2010-2011	NA	NA	0.83	0.75
2011-2012	NA	NA	0.37	0.11
2012-2013	NA	NA	0.91	0.91
