# Supplementary material for: Reassessing Google Flu Trends Data for Detection of Seasonal and Pandemic Influenza: A Comparative Epidemiological Study at Three Geographic Scales
Source: PLoS Comput Biol. 2013 Oct 17;9(10):e1003256. doi: 10.1371/journal.pcbi.1003256 (PMC3798275; doi:10.1371/journal.pcbi.1003256)
Supplement: Table S5 — Influenza season epidemic intensity in New York, 2003–2013. (PDF) [file pcbi.1003256.s012.pdf]

Table S5 – Influenza season epidemic intensity in New York, 2003-2013

New York City, Emergency Department (ED) influenza-like illness (ILI) syndromic surveillance

Epidemic Season	Epidemic period				
	weeks	excess	lower 95%	upper 95%	peak
2003/2004	9	23.87	21.17	26.57	7.54
2004/2005	19	18.66	12.96	24.36	4.89
2005/2006	12	8.19	4.59	11.79	3.27
2006/2007	11	11.34	8.04	14.64	3.50
2007/2008	16	18.71	13.91	23.51	4.59
2008/2009	10	7.24	4.24	10.24	3.30
spring-2009	11	55.51	52.21	58.81	14.25
2009/2010	25	26.46	18.96	33.96	4.49
2010/2011	15	15.44	10.94	19.94	4.38
2011/2012	0	0.00	0.00	0.00	2.58
2012/2013	12	23.31	19.71	26.91	5.91

New York State, Google Flu Trends (GFT) original model

Epidemic Season	Epidemic period				
	weeks	excess	lower 95%	upper 95%	peak
2003/2004	9	26.03	23.51	28.55	8.06
2004/2005	16	18.28	13.80	22.76	3.93
2005/2006	14	6.62	2.70	10.54	2.19
2006/2007	15	9.55	5.35	13.75	2.79
2007/2008	14	16.99	13.07	20.91	4.20
2008/2009	9	7.50	4.98	10.02	3.04
spring-2009	3	1.28	0.44	2.12	1.42
2009/2010	NA	NA	NA	NA	NA
2010/2011	NA	NA	NA	NA	NA
2011/2012	NA	NA	NA	NA	NA
2012/2013	NA	NA	NA	NA	NA

New York State, Google Flu Trends (GFT) updated model

Epidemic Season	Epidemic period				
	weeks	excess	lower 95%	upper 95%	peak
2003/2004	8	27.49	24.85	30.13	9.87
2004/2005	16	21.62	16.34	26.90	4.16
2005/2006	14	8.41	3.79	13.03	2.25
2006/2007	14	7.91	3.29	12.53	2.37
2007/2008	14	26.25	21.63	30.87	5.63
2008/2009	10	11.22	7.92	14.52	3.47
spring-2009	11	17.64	14.01	21.27	3.14
2009/2010	18	36.73	30.79	42.67	6.80
2010/2011	15	15.61	10.66	20.56	3.71
2011/2012	5	1.34	-0.31	2.99	2.72
2012/2013	12	62.31	58.35	66.27	13.05

New York City (experimental), Google Flu Trends (GFT) updated model

Epidemic Season	Epidemic period				
	weeks	excess	lower 95%	upper 95%	peak
2003/2004	9	24.13	20.80	27.46	7.79
2004/2005	15	17.28	11.73	22.83	3.38
2005/2006	11	5.72	1.65	9.79	2.33
2006/2007	7	5.01	2.42	7.60	2.38
2007/2008	13	22.83	18.02	27.64	4.99
2008/2009	8	8.24	5.28	11.20	3.08
spring-2009	12	15.36	10.92	19.80	3.11
2009/2010	15	24.34	18.79	29.89	4.43
2010/2011	12	15.48	11.04	19.92	3.76
2011/2012	5	2.15	0.30	4.00	2.94
2012/2013	21	71.71	63.94	79.48	12.69
