# Supplementary material for: Reassessing Google Flu Trends Data for Detection of Seasonal and Pandemic Influenza: A Comparative Epidemiological Study at Three Geographic Scales
Source: PLoS Comput Biol. 2013 Oct 17;9(10):e1003256. doi: 10.1371/journal.pcbi.1003256 (PMC3798275; doi:10.1371/journal.pcbi.1003256)
Supplement: Table S6 — Google Flu Trends (GFT) model correlation, New York, 2003–2013. (PDF) [file pcbi.1003256.s013.pdf]

Table S6 – Google Flu Trends (GFT) model correlation, New York, 2003-2013

New York City, Emergency Department (ED) respiratory and febrile (R&F) syndromic surveillance

Time Period	original GFT Rsq (observed)	original GFT Rsq (excess)	updated GFT Rsq (observed)	updated GFT Rsq (excess)
Retrospective GFT model training periods	0.89	0.82	0.51	0.44
Prospective GFT model surveillance	0.03	0.03	0.77	0.77
All study weeks	0.34	0.21	0.41	0.33

Time Period	original GFT Rsq (observed)	original GFT Rsq (excess)	updated GFT Rsq (observed)	updated GFT Rsq (excess)
Influenza seasons 2003-2009 (pre-pandemic)	0.87	0.81	0.84	0.79
March 29, 2009 - January 30, 2010	0.31	0.89	0.20	0.29
- pandemic A/H1N1-2009 spring wave	0.78	0.95	0.88	0.86
- pandemic A/H1N1-2009 fall wave	NA	NA	0.27	0.18
Influenza seasons 2010/2011-2011/2012	NA	NA	0.74	0.54
Influenza season 2012/2013	NA	NA	0.92	0.92

Year (June-May)	original GFT Rsq (observed)	original GFT Rsq (excess)	updated GFT Rsq (observed)	updated GFT Rsq (excess)
2003-2004	0.95	0.94	0.96	0.95
2004-2005	0.82	0.65	0.78	0.60
2005-2006	0.83	0.51	0.63	0.27
2006-2007	0.82	0.49	0.84	0.52
2007-2008	0.92	0.85	0.90	0.85
2008-2009	0.03	0.03	0.50	0.66
2009-2010	NA	NA	0.51	0.56
2010-2011	NA	NA	0.74	0.51
2011-2012	NA	NA	0.80	NA
2012-2013	NA	NA	0.95	0.94
