# Supplementary material for: Reassessing Google Flu Trends Data for Detection of Seasonal and Pandemic Influenza: A Comparative Epidemiological Study at Three Geographic Scales
Source: PLoS Comput Biol. 2013 Oct 17;9(10):e1003256. doi: 10.1371/journal.pcbi.1003256 (PMC3798275; doi:10.1371/journal.pcbi.1003256)
Supplement: Text S1 — Technical appendix: Supplementary methods and results. (PDF) [file pcbi.1003256.s014.pdf]

Technical Appendix: Supplementary Methods and Results

Comparison of Google Flu Trends and traditional influenza surveillance highlights the limits of internet search-query data for disease monitoring

Donald R. Olson[1]*, Kevin J. Konty[1], Marc Paladini[1], Cecile Viboud[2], Lone Simonsen[2,3]

1. New York City Department of Health and Mental Hygiene, New York, NY, USA; 2. Fogarty International Center, National Institutes of Health, Bethesda, MD, USA and 3. Department of Global Health, George Washington University, Washington DC, USA;

* Corresponding Author: DRO, dolson@health.nyc.gov; drolson@gmail.com

Data Sources

All analyses in this study are based on the following data: the United States (US) Centers for Disease Control and Prevention (CDC) nationwide and regional influenza-like illness (ILI) sentinel physician network surveillance system ILI-Net [2], the New York City Department of Health and Mental Hygiene (DOHMH) emergency department (ED) syndromic surveillance system [25-27], the original Google Flu Trends (GFT) model estimates for the United States (US) nationwide, by Census Region and by State [13,16], and the updated GFT model estimates for the US nationwide, by Health and Human Services (HHS) Region, State and "experimental" city level [14,16]. All data, analyses and estimates are shown for the National US level (Tables S1-S2; Figures S1-S2), the Mid-Atlantic States (Tables S3-S4; Figures S3-S4), and the State and Local level for New York State and New York City (Tables S5-S6; Figures S5-S7).

National and Regional CDC Surveillance: The national ILI sentinel data used in this study are from the ongoing prospective ILI-Net surveillance network which is coordinated by CDC [2]. For the ten influenza seasons, 2003/04 to 2012/13, the publicly available CDC data provide continuous weekly estimates of ILI for June 2003 through February 2013. The weighted CDC weekly ILI visit proportions are used for national US and Mid-Atlantic Census Region and HHS-2 Region data [2]. Finalized sentinel CDC ILI proportions are used for the 2003/04 through 2011/12 influenza seasons, while provisional surveillance reports for the 2012/13 influenza season provide a preliminary picture based on data updated and available March 30, 2013 [2].

Regional CDC ILI-Net data used in the study are for Mid-Atlantic Census Region States of New Jersey (NJ), New York (NY) and Pennsylvania (PA) for comparison with the original GFT model for the period June 2003 to August 2009. The corresponding regional comparison with the updated GFT model are with the HHS-2 surveillance region which included only NJ and NY. While CDC ILI-Net data are aggregated for both of these regions (Mid-Atlantic and HHS-2), the original GFT model data is available only as Mid-Atlantic states (NJ, NY, PA), and the updated GFT model is available only for HHS aggregation (NJ and NY).

New York City ED Syndromic Surveillance: The local influenza-related ED syndromic surveillance data used in this study are from the ongoing prospective electronic syndromic surveillance system initiated and run by the NYC DOHMH since the autumn of 2001 [25]. The DOHMH ED syndromic system is run on a daily basis, collecting data from participating EDs typically within 24-hours, and covering from 65% of all ED visits in NYC in 2002, to >95% of all ED visits since 2008 [26,27]. The New York City DOHMH began incorporating the ED syndromic surveillance system data for ILI into the CDC sentinel network during the 2007/08 influenza season [39].

Original Google Flu Trends Model: The original GFT model was developed through a process of retrospectively analyzing billions of internet search queries to identify a collection of specific search term strings that correspond with the observed course of ILI in the population, as reported to the U.S. CDC ILI network surveillance system [2]. Original GFT model time periods are for the retrospective training period from September 28, 2003 through March 17, 2007. The prospective GFT model validation period was for March 18, 2007 through May 17, 2008, and ongoing operation, May 18, 2008 through Aug 1, 2009. As reported by Google prospective validation of the original GFT model during the 2007/2008 season found high correlation between GFT estimates and CDC-reported ILI, with next-day timeliness compared to a 1-2 week delay reported in traditional CDC ILI data [13]. The assertion in the original GFT paper, however, that internet search term estimates could enable improved public health response to seasonal and pandemic influenza, did not take into account the practice of modern, electronic syndromic surveillance practice [17]. The publically available estimates from the original GFT model used in this study include data for June 1, 2003 to August1, 2009, accessed on August 1, 2009 [16]. Original GFT model data for August and early-September 2009 are presented in the Google study on the updated GFT model [14], however, they were no longer available in December 2010 when we initiated the current study, and so were not included in our analysis. All original GFT analyses for the Mid-Atlantic Region were compared against the US CDC Census Region Mid-Atlantic States (NJ, NY, PA).

Updated Google Flu Trends Model: The updated GFT model was redefined following the spring and summer wave and initial weeks of the fall wave of the 2009 A/H1N1 influenza pandemic in the United States [14]. Using historical and recent pandemic up-to-date ILI surveillance data from the US CDC for nationwide coverage and by HHS region, the GFT algorithm was recalibrated and a new case-query definition was defined. The updated GFT model time periods for the retrospective query selection training period ran from September 28, 2003 through September 18, 2009. The prospective operation period ran from September 19, 2009 through March 30, 2013 (14). The updated GFT estimates are available at the nationwide, HHS-2 region (NJ, NY), NY state and experimental New York City level.

Comparison of Mid-Atlantic and HHS-2 Regional ILI Surveillance Data: The CDC ILI-Net data are available aggregated by both Census and HHS Regions for the Mid-Atlantic States, while GFT estimates are only available by Census Region (NJ, NY, PA) for the original GFT model, or HHS Region (NJ, NY) for the updated GFT model. Weekly trends and estimates of the timing and intensity for the CDC ILI-Net data for the entire period 2003-2013 are given (Tables S3-S4; Figures S3-S4).

Comparison of GFT estimates for New York State and New York City: The original GFT model estimates were provided at the National, Census Region and State levels only. The updated GFT model estimates are at the US, HHS Region and State levels and select cities in the US. The updated GFT model estimates for both NY State and New York City are compared with each other, and with ILI surveillance data for New York City (the city population comprises 42% of NY State). The observed trends and measured excess epidemic intensity compared between the updated GFT model estimates for NY State and New City are nearly identical: weekly correlations between updated GFT model estimates for New York City and NY State were very highly correlated (R^2>0.98; 2009 pandemic R^2=0.95) (Tables S5-S6; Figures S5-S7).

Case Definitions

Surveillance ILI definitions: National and Regional CDC ILI-Net weighted percent weekly estimates in the study are based on aggregate counts of patients presenting ILI defined as "fever with cough and/or sore throat" in the absence of another cause [2]. The ED syndromic ILI surveillance definition used in the New York City DOHMH system is based on electronic chief complaint records for individual patient visits, classified as 'fever' with 'cough' and/or 'sore throat' sub-syndrome terms [25,26], analogous to the clinical ILI definition used in the CDC ILI-Net system [2].

Internet search case-query definitions: The original GFT model case definition was made up of the top 45 exact search query term text strings selected from the 50 million most common search queries entered by Google users through a simple regression model that analyzed the billions of annual search events during 2003 to 2007 [13]. The original GFT model case-query selection process identified the aggregate weekly scaled search queries that most closely correlated with US

CDC surveillance weighted ILI weekly proportions nationwide and by Census Region [2]. Following the initial spring and summer wave of the 2009 pandemic, the GFT model was updated with surveillance data through the first 20 weeks of the 2009 A/H1N1 pandemic, using a similar regression methodology as the original GFT model, though with a less exclusive qualitative query term selection process which allowed for many additional specific searches to be included in the case definition [14]. Both the original and updated GFT model case-query ILI definition search terms remain undisclosed.

Baseline and Intensity

Estimation of baseline and intensity: A naïve Serfling seasonal regression model was applied to time series of observed ILI surveillance data and GFT search query estimates to calculate baseline expected weekly proportions and 95% epidemic thresholds. Baseline model estimates were calculated using the observed weekly data, with the upper-quartile epidemic and 2009 pandemic weeks removed. Consecutive epidemic period weeks were measured as the sum of weekly excess-ILI proportions for estimates of incident epidemic intensity or magnitude. The baseline Serfling regression confidence intervals represented the upper and lower 95% limit of the non-epidemic baseline, calculated as the predicted baseline +1.96 standard deviations [28-33].

Epidemic timing: Consecutive weeks exceeding the 95% epidemic threshold represented epidemic weeks. The baseline model was fit retrospectively for 2003-2012, with the epidemic periods identified as consecutive weeks exceeding each epidemic threshold and the initial week exceeding the threshold representing the point of detection of the initial epidemic increase. Epidemic period peak weeks were identified as the week reporting the highest ILI proportion. The inherent timing in the time-series data were assessed by cross-correlation, not as a measure of detection but rather as a measure of GFT accuracy compared to gold standard ILI public health surveillance data.

Retrospective and prospective GFT accuracy: Observed and model excess estimates of weekly ILI proportions were compared for GFT models and corresponding national or local surveillance ILI data. Week-to-week correlations for the original and updated GFT models were estimates against corresponding ILI public health surveillance across the three scales: national, regional and local. Cross correlation values were calculated to identify the leading and lagging correspondence between the GFT and public health surveillance data, with maximum correlation values indicating the ideal lead/lag relationship. Model accuracy was also measured as the magnitude of the cumulative epidemic burden measured by GFT compared to the corresponding public health ILI surveillance data.
